# Testicular salvageability and its predictors among patients with testicular torsion in a resource limited setting: a multicentre longitudinal study

**DOI:** 10.1186/s12893-023-02118-z

**Published:** 2023-08-21

**Authors:** Mohamed Abdullahi Mohamed, Demoz Abraha, Anthoney Ayotunde Olasinde, Ahmed Kiswezi, Selamo Fabrice Molen, Joshua Muhumuza, Herman Lule

**Affiliations:** 1 Faculty of Clinical Medicine and Dentistry, Department of Surgery, Kampala International University Western Campus, PO. Box 70, Ishaka-Bushenyi, Uganda; 2 Injury Epidemiology and Prevention Research Group, Division of Clinical Neurosciences, University of Turku, Turku, Finland

**Keywords:** Testicular torsion, Salvageability, Resource limited setting, Uganda

## Abstract

**Introduction:**

Testicular torsion refers to ischemia of the testicle due to twisting or rotation of the vessels supplying the testes. It is a urologic emergency requiring a high index of clinical suspicion and prompt surgical intervention with management aimed at avoiding testicular loss and resulting infertility. This paper gives an update on the current situation regarding this topic in low-income settings. The aim of this study was to determine testicular salvageability and its predictors amongst patients with testicular torsion at two tertiary African hospitals.

**Methods:**

This was a hospital-based multicentre longitudinal study at two tertiary hospitals in western Uganda. Patients with acute scrotum were enrolled and evaluated for testicular torsion. Those with confirmed testicular torsion underwent surgery and salvageability was reported as the primary outcome. Predictors for testicular salvageability were determined using backward binary logistic regression in SPSS version 22.

**Results:**

During the study period, 232 patients with acute scrotum were enrolled. The mean age was 35.3 (SD = 20.4) years. Forty-one (17.7%) patients had testicular torsion. Only 16 (39.0%) of patients with torsion had viable testes that were salvageable. Orchiectomy was performed on 25 patients (61.0%). At multivariate analysis, a patient who presented after 48 h from the onset of symptoms was 34.833 times more likely to have orchiectomy compared to one who presented within 12 h [AOR = 34.833, (95% CI = 5.020-60.711), P < 0.001].

**Conclusion:**

In this study, the testicular salvage rate was low. The only predictor of salvageability was the time from the onset of symptoms to presentation. All males should be sensitized about the clinical features of testicular torsion to ensure early presentation to increase salvage rates.

**Supplementary Information:**

The online version contains supplementary material available at 10.1186/s12893-023-02118-z.

## Background

Testicular torsion refers to ischemia of the testicle due to twisting or rotation of the vessels supplying the testis, through the lengthwise of the spermatic cord presenting as an acute scrotum [1]. The classic presentation of testicular torsion is a sudden onset of severe unilateral pain, associated with nausea and vomiting; often with referred pain to the ipsilateral iliac fossa [2]. The examination findings include: a high riding testis with an abnormal transverse lie, a thickened spermatic cord, an absent cremasteric reflex, as well as erythema, tenderness, and swelling [2]. Ultrasonography and Doppler’s study have been regarded to be highly reliable and beneficial in avoiding unnecessary surgical exploration with sensitivity and specificity of 86 to 100% and 95–100% respectively; although their roles have been criticized concerning the accuracy, availability, cost, delay in treatment, and operator-dependence [3].

The acutely painful scrotum has been described as a urologic emergency, requiring a high index of clinical suspicion, and prompt surgical intervention with the management aimed at avoiding testicular loss and infertility [1]. Usually, salvage rates depend on the duration of symptoms and speed of diagnosis [4]. A study reported that the salvage rate is high if the treatment is initiated within six hours after the onset of symptoms, and inversely decreases with the increase in duration of symptoms [5]. However male health seeking behavior, long commute distances, economic constraints and inadequate access to specialist urological care can potentially prohibit timely presentation in low income settings.

The annual incidence of testicular torsion is approximately 3.8 per 100,000 in the general population [6]. Testicular torsion presents a diagnostic challenge due to the overlap of its clinical presentation with that of epididymitis and torsion of appendix testis [7]. Though testicular torsion is a surgical emergency that warrants operation within 6 h of onset [8], epididymitis and torsion of the appendix testis do not require such urgency. Patients in resource limited settings often present late as they wait for the pain to resolve with self-medication. Moreover, clinicians may prescribe antibiotics and analgesics when a misdiagnosis has been made, resulting in late presentation [4]. Generally, in our setting, it’s unlikely for a patient to present within six hours due to long travel distances to access surgical specialty services and the tendency to use alternative medicine. A study in Uganda at the National Referral Hospital revealed that, over 50% of the patients with testicular torsion reported to the hospital after 48 h of onset of symptoms, and consequently; only 25% of the testis were salvaged [9]. Given the high rates of testicular loss following torsion due to poor diagnostics and referral systems in Africa, there is a need for an update on the current situation regarding this subject. Thus, the aim of this study was to determine testicular salvageability and its predictors amongst patients with testicular torsion at two tertiary Ugandan hospitals.

## Study methods

### Study design, setting and population

This was a hospital-based prospective observational cohort study simultaneously conducted at Kampala International University Teaching Hospital (KIUTH) and Hoima Regional Referral Hospital (HRRH). Data collection lasted 6 months (August 2022 to January 2023). Both hospitals have surgical, laboratory and imaging departments. In these hospitals, cases of acute scrotum are managed by general surgeons due to the unavailability of urologists.

### Eligibility criteria and sample size estimation

We included all patients who presented with acute scrotum except those with a history of trauma.

Sample size was determined using Daniel’s formula $n=\frac{Z^{2} \times Pq}{{e}^{2}}$, [10]. Using the findings of Opio and colleagues who reported that testicular torsion was seen in 16.4% of the patients with acute scrotum at Mulago National Referral Hospital in Uganda [9], n=210.7. By adding 10% to increase internal validity and cater for loss of follow-up, the minimum sample size required for this study was 232 participants.

### Recruitment and data collections procedure

Eligible patients were consecutively recruited into the study at the accident and emergency department or surgical ward until the required sample size was reached. Informed consent was obtained from all participants prior to recruitment. Clinical assessment was performed in addition to urinalysis, and Doppler ultrasonography to assess blood flow to the testes. Participants with suspected or confirmed testicular torsion underwent surgical exploration.

### Study variables

During history taking and clinical examination, the following information was obtained: age, residence, education level, monthly income, time to presentation, referral status, presence of pain, scrotal swelling, nausea, vomiting, loss of the cremasteric reflex, trauma history, fever, hypertension, tachycardia, tachypnea, and peripheral oxygen saturation in addition to determining the TWIST score.

Urinalysis by use of dip stick and microscopy of mid-stream urine samples was performed by a qualified laboratory technician. Patients underwent scrotal Doppler sonography that was performed by a qualified radiologist. The Doppler sonography procedure was adopted from Agrawal et al., [11], in which the patients were subjected to high frequency ultrasonography and color Doppler using high resolution and color Doppler linear probe (7.5–12 MHz). Serial transverse and sagittal images of each scrotum were obtained and both testes were compared for echotexture and color flow. The study included both the scrotum and inguinal area. Results were dichotomized as compromised blood supply or not compromised for simplicity of analyses, although detailed reports were availed to attending clinicians to inform surgical decisions.

The diagnosis of testicular torsion was done on the basis of history of recent sex, trauma, pain, nausea, and vomiting, clinical signs of elevated tender testicle, twist criterion [8], and intra operative findings. To clinically assess the severity of testicular torsion, the Testicular Workup for Ischemia and Suspected Torsion (TWIST) score has been developed to risk stratify testicular torsion in males presenting with an acute scrotum (AS). The TWIST score is based on the sum (ranging from 0 to 7) of the following historical and physical examination findings: testicular swelling (2 points), hard testicle (2 points), absent cremasteric reflex (1 point), nausea or vomiting (1 point), and high riding testicle (1 point).The risk stratification scores for patients at low risk for testicular torsion is 0 to 2 points, the intermediate risk is 3 to 4 points, and high risk for testicular torsion is 5 to 7 points [8].

The routine management for other diagnosed conditions other than testicular torsion included emergency open hernia repair for complicated inguinal scrotal hernias, administration of antibiotics and non-steroidal anti-inflammatory medication for epididymitis/orchitis, incision and drainage for scrotal abscess and referral to a urologist for testicular cysts and undiagnosed scrotal masses.

The surgery for testicular torsion was performed by qualified surgeons. The participants were routinely reviewed during ward rounds until they were discharged from the hospitals. The research team ensured completeness of the questionnaire before discharge.

### Quality control and data analysis

The questionnaire was pretested, and necessary changes were made before starting data collection. The principal investigator and trained research assistants collected the data. Sonography was performed by a qualified radiologist. Data was checked for completeness at the end of each entry and the analyses were performed with the guidance of a biostatistician.

Data were summarized and analyzed using Statistical Package for the Social Sciences (SPSS Inc., Chicago, USA, version 22.0 for Windows). The proportion of patients confirmed to have torsion among all presenting with acute scrotum was computed with a corresponding 95% confidence interval. Salvageability was computed as the percentage of patients in whom the testis survived of all patients with testicular torsion. Bivariate analysis was conducted and the variables with p-values of ≤ 0.2 in bivariate analysis and those factors with biological plausibility were considered for multivariate analysis. Those variables with p-values of ≤ 0.05 in the stepwise backward binary logistic regression analysis were regarded to be statistically significant predictors of salvageability. Binary logistic regression was used because salvageability had two possible outcomes. Throughout the analyses, reference groups were selected based on a category presumed to have a minimal or no risk for the outcome of interest (i.e., categories closely representing normal population).

## Results

During the study period, 232 patients with acute scrotum were enrolled. The mean age was 35.3 (SD = 20.4) years. The majority of the participants came from rural areas 154 (66.4%) and were admitted at Hoima regional referral hospital 196 (84.5%). Of the 232, only 41 (17.7%, CI = 13.4-22.4%) had testicular torsion (Table 1).

**Table 1:** Baseline characteristics of study participants

Characteristic	Statistic	
Age in years	Mean = 35.3, SD = 20.4, Min = 0.06, Max = 86.0.	
	**Frequency**	**Percentage**
WHO Age category		
0–16	39	16.8
17–30	66	28.4
30–45	61	26.3
> 45	66	28.4
Residence		
Rural	154	66.4
Urban	78	33.6
Education level		
No Formal Education	89	38.4
Primary	50	21.6
Post Primary	93	40.1
Monthly Income (UGX)		
< 500,000	106	45.7
500,000–1 Million	100	43.1
> 1 million	26	11.2
Hospital		
HRRH	196	84.5
KIU-TH	36	15.5
Diagnosis		
Complicated ISH	149	64.2
Torsion	41	17.7
Epididymitis/orchitis	27	11.6
Others (*Testicular cyst, Scrotal mass, scrotal abscess*)	15	6.5

SD = Standard deviation, Min = Minimum, Max = maximum, HRRH = Hoima regional referral hospital, KIU-TH = Kampala international university teaching hospital, ISH = Inguinal scrotal hernia, UGX = Uganda shillings

### Testicular salvageability and its ppredictors

Forty-one patients were diagnosed with torsion, and of these, only 16 (39.0%, CI = 24.4-53.7%) had viable testes that were salvaged. Orchiectomy was performed on 25 patients (61.0%, CI = 46.3-75.6%). The results of the bivariate analysis are shown in Table 2. At bivariate analysis, the variables that had a p value less than 0.2 and therefore qualified for multivariate analysis were time to presentation of symptoms 13–24 h (cOR = 11.000, CI = 0.646-187.166, P = 0.097), 25–48 h (cOR = 11.000, CI = 0.646-187.166, P = 0.097), > 48 h (cOR = 34.833, CI = 5.020-241.711, P < 0.001), loss of cremasteric reflex (cOR = 4.000, CI = 1.000-15.994, P = 0.050), presence of tachycardia (cOR = 11.786, CI = 1.342-103.515, P = 0.026) and the testicular workup for ischemia and suspected torsion (TWIST) score category (cOR = 5.538, CI = 0.522–58.756, P = 0.155).

**Table 2:** Bivariate analysis of association between testicular salvageability and the independent variables

Characteristic	Salvaged N = 16 n (%)	Orchiectomy N = 25 n (%)	Bivariate analysis		
			**cOR**	**95% CI**	**P value**
WHO Age category					
0–16	7(17.1)	15(36.6)	Ref		
17–30	8(19.5)	7(17.1)	0.408	0.105–1.582	0.195
30–45	1(2.4)	3(7.3)	1.400	0.123–15.974	0.786
Residence					
Rural	11(26.8)	19(46.3)	1.439	0.355–5.837	0.610
Urban	5(12.2)	6(14.6)	Ref		
Education level					
Non-Formal	9(22.0)	11(26.8)	1.222	0.291–5.128	0.784
Primary	1(2.4)	8(19.5)	8.000	0.750-85.313	0.085
Post Primary	6(14.6)	6(14.6)	Ref		
Monthly income					
< 500,000	7(17.1)	12(29.3)	0.843	0.239–2.975	0.790
500,000- 1 M	9(22.0)	13(31.7)	Ref		
Time to presentation (in hours)					
0–12	11(26.8)	2(4.9)	Ref		
13–24	1(2.4)	2(4.9)	11.000	0.646-187.166	**0.097**
25–48	1(2.4)	2(4.9)	11.000	0.646-187.166	**0.097**
> 48	3(7.3)	19(46.3)	34.833	5.020-241.711	**< 0.001**
Referred from health facility					
Yes	6(14.6)	13(31.7)	1.806	0.502–6.498	0.366
No	10(24.4)	12(29.3)	Ref		
Referred from an academic institution					
Yes	7(17.10	11(26.8)	1.010	0.285–3.578	0.987
No	9(22.0)	14(34.1)	Ref		
From Home					
Yes	5(12.2)	8(19.5)	1.035	0.268–3.995	0.960
No	11(26.8)	17(41.5)	Ref		
Scrotal swelling					
Yes	15(36.6)	23(56.1)	0.767	0.064–9.220	0.834
No	1(2.4)	2(4.9)	Ref		
Nausea/Vomiting					
Yes	15(36.6)	20(48.8)	0.267	0.028–2.527	0.249
No	1(2.4)	5(12.2)	Ref		
Loss of Cremasteric reflex					
Yes	8(19.5)	20(48.8)	4.000	1.000-15.994	**0.050**
No	8(19.5)	5(12.2)	Ref		
Trauma history					
Yes	7(17.1)	9(22.0)	0.723	0.201–2.605	0.620
No	9(22.0)	16(39.0)	Ref		
Fever history					
Yes	4(9.8)	6(14.6)	0.947	0.221–4.067	0.942
No	12(29.3)	19(46.3)	Ref		
Hypertension					
Yes	1(2.4)	3(7.3)	2.045	0.194–21.586	0.552
No	15(36.6)	22(53.7)	Ref		
Tachycardia					
Yes	1(2.4)	11(26.8)	11.786	1.342-103.515	**0.026**
No	15(36.6)	14(34.1)	Ref		
Tachypnea					
Yes	10(24.4)	13(31.7)	0.650	0.181–2.339	0.510
No	6(14.6)	12(29.3)	Ref		
Other symptoms					
None	7(17.1)	9(22.0)	Ref		
Dysuria	4(9.8)	5(12.2)	0.972	0.188–5.034	0.973
Others	5(12.2)	11(26.8)	1.711	0.403–7.271	0.467
Compromised blood supply on Doppler					
Yes	7(17.10	11(26.8)	1.010	0.285–3.578	0.987
No	9(22.0)	14(34.1)	Ref		
Urinalysis					
Normal	10(24.4)	17(41.5)	Ref		
Abnormal	6(14.6)	8(19.5)	0.784	0.210–2.923	0.717
Anemia					
Yes	7(17.1)	15(36.6)	1.929	0.541–6.875	0.311
No	9(22.0)	10(24.4)	Ref		
TWIST Score					
2–4	3(7.3)	1(2.4)	Ref		
> 4	13(31.7)	24(58.5)	5.538	0.522–58.756	**0.155**

Ref = Reference category, cOR = Crude odds ratio, CI = Confidence interval

The results of the multivariate analysis are shown in Table 3. In multivariate analysis, the time to presentation of symptoms was the only significant predictor of salvageability. A patient who presented after 48 h from the onset of symptoms was 34.8 times more likely to have orchiectomy compared to one who presented within 12 h (AOR = 34.833, CI = 5.020-60.711, P < 0.001).

**Table 3:** Multivariable analysis of association between testicular salvageability and the independent variables

Characteristic	Multivariate analysis		
	**AOR**	**95% CI**	**P value**
Time to presentation (in hours)			
0–12	Ref		
13–24	11.000	0.646–57.166	0.097
25–48	11.000	0.646–57.166	0.097
> 48	**34.833**	**5.020-60.711**	**< 0.001**
Loss of Cremasteric reflex			
Yes	3.935	0.287–53.876	0.305
No	Ref		
Tachycardia			
Yes	2.750	0.211–35.838	0.440
No	Ref		
TWIST Score			
2–4	Ref		
> 4	2.436	0.028-212.302	0.696
Age category			
0–16	Ref		
17–30	0.968	0.053–17.841	0.982
30–45	0.600	0.027–13.582	0.748

AOR = Adjusted odds ratio, Ref = Reference category, CI = Confidence interval

## Discussion

In this study, of the 41 patients that were diagnosed with torsion, only 16 (39.0%, CI = 24.4-53.7%) had viable testes that were salvaged. Orchiectomy was performed for 25 (61.0%, CI = 46.3-75.6%). Our findings are comparable to what was reported at Mulago Hospital in Uganda, where 63.7% of the patients with testicular torsion underwent orchiectomy [9]. Since the upper limit of our confidence interval was 75.6%, there was no significant difference between the percentages of patients that underwent orchiectomy in the Mulago study compared to our study. Also, in agreement with our findings is a systematic review that reported salvage rates ranging from 14 to 81% [12].

Contrary to our findings, multiple studies in high-income countries reported much lower percentages of orchiectomy including: 42% in Iowa [13], 28% in Canada [14] and 40% in Jordan [15]. Studies that reported significantly higher rates of orchiectomy include one in Kenya with 82% [4], and another in Ethiopia where 83% had orchiectomy [12].

Overall, it can be noted that lower salvage rates were reported in low-income countries while higher salvage rates were reported in high-income countries. This can be explained by the differences in the health care systems which could have affected the time from onset of symptoms to diagnosis, and from diagnosis to intervention. In addition, variations in levels of awareness and health-seeking behaviors could result in delayed presentation in low-income settings.

The wide variations in the rates of salvage among patients with torsion can be explained by the time variations from the onset of symptoms to presentation. In our study with low salvage rates, the majority (53.7%) of the patients with testicular torsion presented after 48 h. Evidence suggests that presentation after 48 h reduces the chance of testicular salvage to 25% [3]. According to the literature, when twisting takes more than 6–8 h duration, the blood supply is interrupted and cellular death ensues, necessitating the removal of the dead testis [8].

Further, the variations in age of the participants in the different studies could explain the difference in salvage rates. Compelling evidence from literature suggest that neonates have a salvage rate of about 9% [16], and in our study, we had only one neonate.

In this study, a patient who presented after 48 h from the onset of symptoms was 34.8 times more likely to have orchiectomy compared to the one who presented within 12 h (P < 0.001). This agrees with what has been reported in other studies. According to a systematic review by Derbew and Laytin [12], testicular torsion is a surgical emergency and requires prompt surgical exploration and management. Once the decision for surgical management is made, it should occur as quickly as possible. Testicular salvage rates are closely associated with the duration of ischemia, and with a “golden” window of 4 to 8 h from the time of torsion to the time of detorsion [17] due to the nature of blood supply which are end arteries.

According to a literature review by Prendergast and colleagues, intervention for torsion is time-sensitive where a 97% salvage rate in the first 6 h decreases to 61.3% beyond 12 h [18]. At bivariate analysis, loss of cremasteric reflex and presence of tachycardia were associated with a p value less than 0.05 but were not statistically significant at the multivariate level. The association seen at bivariate analysis between the risk of orchiectomy and loss of cremasteric reflex is possibly due to the worsening ischemia as the duration of torsion increases. Further, we believe that the association between tachycardia and the risk of orchiectomy could arguably be due to the increased release of inflammatory mediators as the testis gets ischemic and gangrenous.

### Study limitations

This study was not without limitations. First, considering 12 h as opposed to 6 h presentation as reference in the analyses is reasonable in low-income settings but limits the generalizability of our findings in the context of “late presentation” in the high income countries. Secondly, in this study, age was not significantly associated with testicular salvageability as was seen in other studies [16]. This is possibly because this study had only one neonate, thus a small representation to demonstrate validity of this association. Lastly hematological parameters such as monocyte count which could be a potential predictor for testicular salvageability was beyond the scope of this study.

### Study strengths and generalizability of the findings

This study captured a wide range of age groups and used the color Doppler and intraoperative findings which could be considered the gold standard in our settings. Thus, we believe that our findings are a true representative of burden of testicular torsion and its outcomes in low-middle income countries where patients tend to present late. However, the findings could differ in high income countries where patients present relatively early.

## Conclusion

In this study, the salvage rate was low in comparison to existing literature. The only factor that predicted salvageability was the time from the onset of symptoms to presentation. These results underpin the need for increased sensitization and awareness campaigns amongst all males regarding the early clinical features of testicular torsion to ensure timely presentation to increase the salvage rates.

### Electronic supplementary material

Below is the link to the electronic supplementary material.

Additional File 1: Supplementary file.sav

## Data Availability

Data from this study is available as supplemental material.

## Declarations

### Guarantors

MAM, HL.

## Abbreviations

KIUTH: Kampala International University Teaching Hospital
HRRH: Hoima Regional Referral Hospital
